# Zone-dependent acute circulatory changes in abdominal organs and extremities after resuscitative balloon occlusion of the aorta (REBOA): an experimental model

**DOI:** 10.1186/s40001-021-00485-y

**Published:** 2021-01-21

**Authors:** Sascha Halvachizadeh, Ladislav Mica, Yannik Kalbas, Miriam Lipiski, Marko Canic, Michel Teuben, Nikola Cesarovic, Zoran Rancic, Paolo Cinelli, Valentin Neuhaus, Hans- Christoph Pape, Roman Pfeifer

**Affiliations:** 1 Department of Trauma, University of Zurich, University Hospital Zurich, Raemistrasse 100, 8091 Zurich, Switzerland; 2 Division of Surgical Research, University Hospital Zurich, University of Zurich, Zurich, Switzerland; 3 Department of Vascular Surgery, University Hospital Zürich, Raemistrasse 100, Zurich, Switzerland

**Keywords:** REBOA, Abdominal organ perfusion, Shock, Trauma, Resuscitative balloon occlusion of the aorta, Polytrauma, Emergency intervention

## Abstract

**Introduction:**

Resuscitative endovascular balloon occlusion of the aorta (REBOA) may be used in severely injured patients with uncontrollable bleeding. However, zone-dependent effects of REBOA are rarely described. We compared the short-term zone- and organ-specific microcirculatory changes in abdominal organs and the extremity during occlusion of the aorta in a standardized porcine model.

**Methods:**

Male pigs were placed under general anesthesia, for median laparotomy to expose intra-abdominal organs. REBOA placement occurred in Zone 1 (from origin left subclavian artery to celiac trunk), Zone 2 (between the coeliac trunk and most caudal renal artery) and Zone 3 (distal most caudal renal artery to aortic bifurcation). Local microcirculation of the intra-abdominal organs were measured at the stomach, colon, small intestine, liver, and kidneys. Furthermore, the right medial vastus muscle was included for assessment. Microcirculation was measured using oxygen-to-see device (arbitrary units, A.U). Invasive blood pressure measurements were recorded in the carotid and femoral artery (ipsilateral). Ischemia/Reperfusion (I/R)-time was 10 min with complete occlusion.

**Results:**

At baseline, microcirculation of intra-abdominal organs differed significantly (*p* < 0.001), the highest flow was in the kidneys (208.3 ± 32.9 A.U), followed by the colon (205.7 ± 36.2 A.U.). At occlusion in Zone 1, all truncal organs showed significant decreases (*p* < 0.001) in microcirculation, by 75% at the colon, and 44% at the stomach. Flow-rate changes at the extremities were non-significant (n.s). During occlusion in Zone 2, a significant decrease (*p* < 0.001) in microcirculation was observed at the colon (− 78%), small intestine (− 53%) and kidney (− 65%). The microcirculatory changes at the extremity were n.s. During occlusion in Zone 3, truncal and extremity microcirculatory changes were n.s.

**Conclusion:**

All abdominal organs showed significant changes in microcirculation during REBOA. The intra-abdominal organs react differently to the same occlusion, whereas local microcirculation in extremities appeared to be unaffected by short-time REBOA, regardless of the zone of occlusion.

## Introduction

Following traumatic brain injuries, acute traumatic hemorrhage is the main causes of early death, after severe blunt trauma [1]. Although hemorrhagic control by external pressure, tourniquet [2], or open surgery [3] are the commonly used interventions, endovascular techniques have recently gained considerable more acceptance. Similarly, open procedures for acute bleeding control have decreased as endovascular catheter-based techniques have continued to demonstrate efficacy and durability [4]. The use of endovascular techniques for managing traumatic vascular lesions in solid organ injury is, therefore, gaining greater popularity [5]. At the turn of the century, an approximate 30-fold increase in the use of endovascular techniques in trauma was observed [6], with approximately 13.2% of all blunt vascular injuries treated using endovascular methods [7]. The use of resuscitative endovascular balloon occlusion of the aorta (REBOA) as a modern clinical practice, adds a promising adjunct to the acute treatment of major blood loss in the abdomen or the pelvis [8], with successful elevation of central blood pressure during shock [9]. REBOA also serves as a hemorrhage control and resuscitation adjunct to prevent cardiovascular collapse [9]. Depending on the bleeding source, REBOA may be performed at three different zones: Zone 1 ranges from the left subclavian artery to the coeliac trunk; Zone 2 ranges from the celiac trunk to the most caudal renal artery, and Zone 3 extends from the most caudal renal artery to the aortic bifurcation [8, 9]. There are still discussions on the duration of REBOA. Studies included in a systematic review [9] presented REBOA occlusion times of 63 min (Inter-Quartile-Range (IQR) 33–88 min) in zone 1, and 45 min (IQR 30–105 min) in zone 3, including patients with occlusion times up to 6 and 10 h. Thus, REBOA increases resuscitation times, preventing hemorrhaging by up to 60 min [8, 10].

The introduction of REBOA has led to a growing number of experimental studies, assessing different parameters on REBOA [11]. However, acute organ-specific microcirculatory changes during REBOA have not yet been described. Therefore, in this study we aimed to analyze zone-depending microcirculatory changes in abdominal organs and the extremity during REBOA. We hypothesized that REBOA at different zones would influence regional ischemia–reperfusion changes in intra-abdominal organs.

## Methods

### Ethical statement

Animal housing, and all experimental procedures were in accordance with Swiss animal welfare protection law, and conformed to the European Directive 2010/63/EU of the European Parliament, the Council on the Protection of Animals used for Scientific Purposes, and the Guide for the Care and Use of Laboratory Animals [12]. This study was conducted under license number 86/2019.

### Experimental subjects

This study was conducted in male Swiss pigs (*sus scrofa*) from a local farmer. All animals were pre-medicated with an intramuscular injection of ketamine at 15 mg/kg (Ketamin-Ketasol ® 100, Dr. E. Graeub AG, Bern, Switzerland), midazolam at 0.5 mg/kg (Midazolam-Dormicum ®, Roche Pharma Schweiz AG, Reinach, Switzerland) and atropine at 0.05 mg/kg (Atropinsulfate, kantonal pharmacy Zurich, University Hospital Zurich, Switzerland). General anesthesia was maintained with a combination of propofol (Propofol-® Lipuro, B. Braun Medical AG, Sempach, Switzerland; 5–10 mg/kg/h Constant Rate Infusion (CRI)) and sufentanil forte (Sufenta® Forte, Janssen-Cilag AG, Zug, Switzerland; 0.01 mg/kg/h CRI). Throughout experiments, animals were maintained on a respirator by positive pressure ventilation, with an inspired oxygen fraction (FiO_2_) of 30%, tidal volume of 8–10 ml/kg, a frequency of 15–20 breaths per minute, and a positive end expiratory pressure (PEEP) of 3–5 cm H_2_O. Arterial blood was drawn at the beginning and the end of the experiment for standard arterial blood gas analysis (aBGA) using Epoc Blood Analysis System (Siemens Healthineers Headquarters, Siemens Healthcare GmbH, Herlanken, Germany). Animals received heparin (Heparin-Na 25.000 units/5 ml, B. Braun, Switzerland) throughout the experiment to prevent micro-embolization. We started prophylaxis with 10.000 units of heparin and monitored the activated clotting time (ACT). The ACT was targeted at 200 s throughout the experiment. The reason for the use of heparin was to minimize micro-thrombi that might alter circulatory measurements.

### Outcome measures

Outcome measures included systemic circulatory changes: invasive blood pressure was used to measure mean arterial pressure (MAP) at the right carotid artery (carotis, proximal to the balloon occlusion), and the left femoral artery (femoral, distal to the balloon occlusion). Heart rate was monitored with standard elector-cardiogram (ECG).

During the study and the occlusion of each zone, the following baseline parameters were collected: vital parameters including blood pressure, heart rate, arterial oxygen saturation (SaO_2_), and microcirculation (A.U) including local flow rate (µFlow), local relative hemoglobin (rHb) and local oxygenation levels. At baseline and at study end, experiment blood gas analyses were performed, and local oxygen delivery [A.U.] was calculated using the following formula [13, 14]:

$$\text{Oxygen Delivery }=\text{ Sa}{{\text{O}}_{\text{2}}}\times \text{ rHb }\times \text{ 1}.\text{34 }\times \text{ }\mu \text{Flow}$$

Each organ of interest was measured for 60 s. Regional blood flow was measured using the oxygen to see device (O2C, Lea Inc., Germany). Measures of local microcirculation include: (1) local flow rate, a measure for blood flow, (2) relative hemoglobin levels, representing the local hemoglobin amount, (3) local oxygen saturation, oxygen delivery and oxygen consumption in arbitrary units (A.U), as described previously [15]. In brief, the measurements of this device base on a combined application of two well-established techniques: laser-Doppler-spectroscopy (formerly used in the OptoFlow) and white light tissue spectrometry for determination of oxygen saturation and hemoglobin amount. Movement of erythrocytes cause a Doppler shift that is detected by laser light and analyzed by the O2C. The white light detects hemoglobin parameter including oxygen saturation (SaO_2_) and relative amount of hemoglobin (rHb). Measurements of SaO_2_ base on the changing color of hemoglobin as it is saturated with oxygen. The absorption of light by the tissue represents the hemoglobin value (the greater the amount of blood, the more light will be absorbed). This measurement represents a hemoglobin amount per tissue volume and is independent from the vessel density, vessel lumen and hemoglobin quantity in the blood.

### Surgical methods

A median laparotomy was performed to access abdominal organs including stomach, colon, small intestine, liver and left kidney. A small incision was performed above the medial vastus muscle to access the extremity, for local microcirculatory measures. Local microcirculation in organs was measured on the superficial aspect of the organ, without injuring the organ. Prior to balloon inflation, diagnostic angiography confirmed normal patency of the celiac trunk, superior mesenteric, and both renal arteries in all animals. No signs of stenosis or any other alterations were observed. All animals were kept on a warming pad and the temperature was monitored continuously.

#### REBOA

All animals were placed in a supine position. Access to the femoral artery was prepared using a 14.0Fr, 450 mm sheath. We used a long sheet to support the inflated balloon, preventing it from being pushed distally by the pulsatile blood pressure in the aorta. The REBOA-catheter (Reliant Stent Graft Balloon Catheter, Medtronic, Ireland) was placed through the sheath, through the right femoral artery. The balloon was inflated with contrast agent (Ultravist ® 200, Bayer Vital GmbH, Leverkusen, Germany) (diluted 1:1 in sterile saline solution) to radiologically verify positioning. Complete occlusion of the balloon was radiologically verified by changing shape form round to rectangular indicating the balloon outer edges to be parallel to the aortic wall [16].

After baseline measurements, the balloon was occluded in Zone 3 (between the inferior mesenteric artery and iliac branches) for 10 min. At steady state, measurements were taken and the balloon was deflated. Reperfusion time was set at 10 min. Next, the balloon was inflated in Zone 2 (between the celiac trunk and the lowest renal arteries). After 10 min inflation, and 10 min reperfusion, the balloon was occluded in Zone 1 (above the diaphragm in the descending aorta) [17] A scheme of the anatomical zone location is provided in "Appendix". Time-point “End” was reached 60 min after the first balloon occlusion of the aorta just prior to study termination. The occlusion time of 10 min was chosen for the following reasons: first, the aim of this experiment was to assess the acute microcirculatory changes during REBOA. Secondly, in pigs, it remains unclear after what time organ damages are irreversible or how much time for physiologic reperfusion is required. Therefore, we decided to set the ischemia–reperfusion time at each 10 min so no secondary organ damages interfere with microcirculatory measurements.

### Termination

Animals were terminated after the experiment with Sodium-pentobarbital (Esconarkon ad us. Vet., Streuli Pharma AG, Uznach, Switzerland).

### Statistics

Descriptive statistics of baseline characteristics included the mean, standard deviation (± SD), ranges for continuous variables, median and interquartile ranges for ordinal or non-normal variables, and numbers and percentages of categorical variable totals. Comparisons of between-group differences were calculated, including two-sided 95% confidence intervals (CI), student t tests for continuous variables, and Pearson chi-square tests for discrete variables. Binary secondary outcomes were reported as between-group treatment differences, with 95% CI. Multiple group comparisons were performed using ANOVA. Non-symmetrical distributed variables were compared between groups using an unpaired two-sided Wilcoxon test. Within animal comparisons were performed using Wilcoxon signed-rank tests. All statistical analyses were performed using R (R Core Team (2019). R is a language and environment for statistical computing (R Foundation for Statistical Computing, Vienna, Austria. URL https://www.R-project.org/.) All graphical analyses were performed using GraphPad Prism (version 8.0.0 for Windows, GraphPad Software, San Diego, California USA, www.graphpad.com). Statistical significance was assumed at a *p* value below 0.05.

## Results

Six animals were included in the study. All animals were included in the analyses. The mean weight was 48.6 kg (± 1.9 kg, range 47.0–52.5 kg). The baseline MAP was 77.8 mmHg (± 13.3 mmHg) and heart rate was 81.8 beats per minute (bpm) (± 8.9 bpm). Mean arterial blood pressure was measured in carotid and femoral arteries, and did not differ significantly (95% CI − 0.31 to 12.0, *p* = 0.62). Baseline/physiological flow differed significantly between organs (*p* < 0.001). Microcirculatory measures at the kidneys showed the highest baseline blood-flow (208.3 A.U., ± 30.5 A.U). The extremity showed the lowest blood-flow (67 A.U., ± 22.1 A.U.), significantly lower, compared with abdominal organs (95% CI − 134.4 to − 88.9, *p* < 0.001). The blood-flow of the colon was significantly higher than the stomach (95% CI − 51.9 to − 19.1, *p* < 0.001), and the small intestine (95% CI − 36.1 to − 2.9, *p* = 0.022). The blood-flow of the liver was significantly lower than the kidney (95% CI − 30.2 to  − 3.2, *p* = 0.016).

### Changes during REBOA

#### Changes during occlusion in Zone 1

**Fig. 1:**
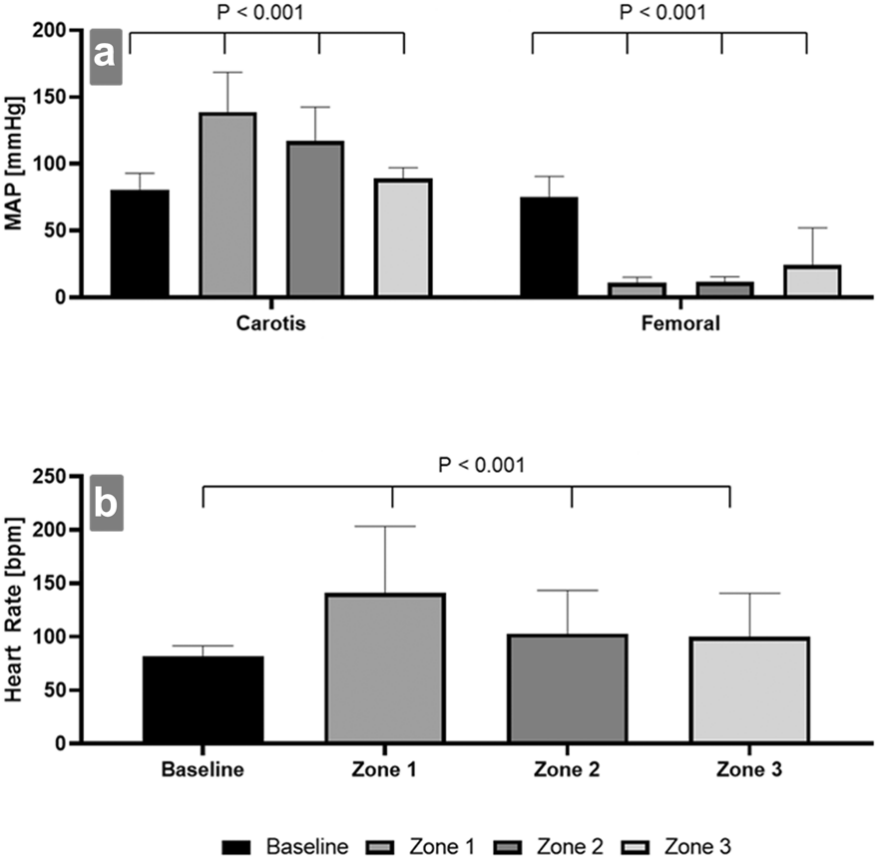
**a** Occlusion-zone-dependent changes in the mean arterial pressure (MAP) measured at the carotic artery (carotis, proximal to the occlusion) and femoral artery (femoral, distal to the occlusion). The MAP increased significantly when compared to baseline. The increase was highest during occlusion in zone 1, followed by zones 2 and 3. The MAP distal to the balloon dropped significantly when compared to baseline. The MAP decrease at the femoral artery was strongest during occlusion in zones 1 and 2, followed by 3. **b** Occlusion-zone-dependent changes in heart rate (beats per minute, (bpm)). Heart rate increased significantly during occlusion in zone 1, followed by zones 2 and 3

**Fig. 2:**
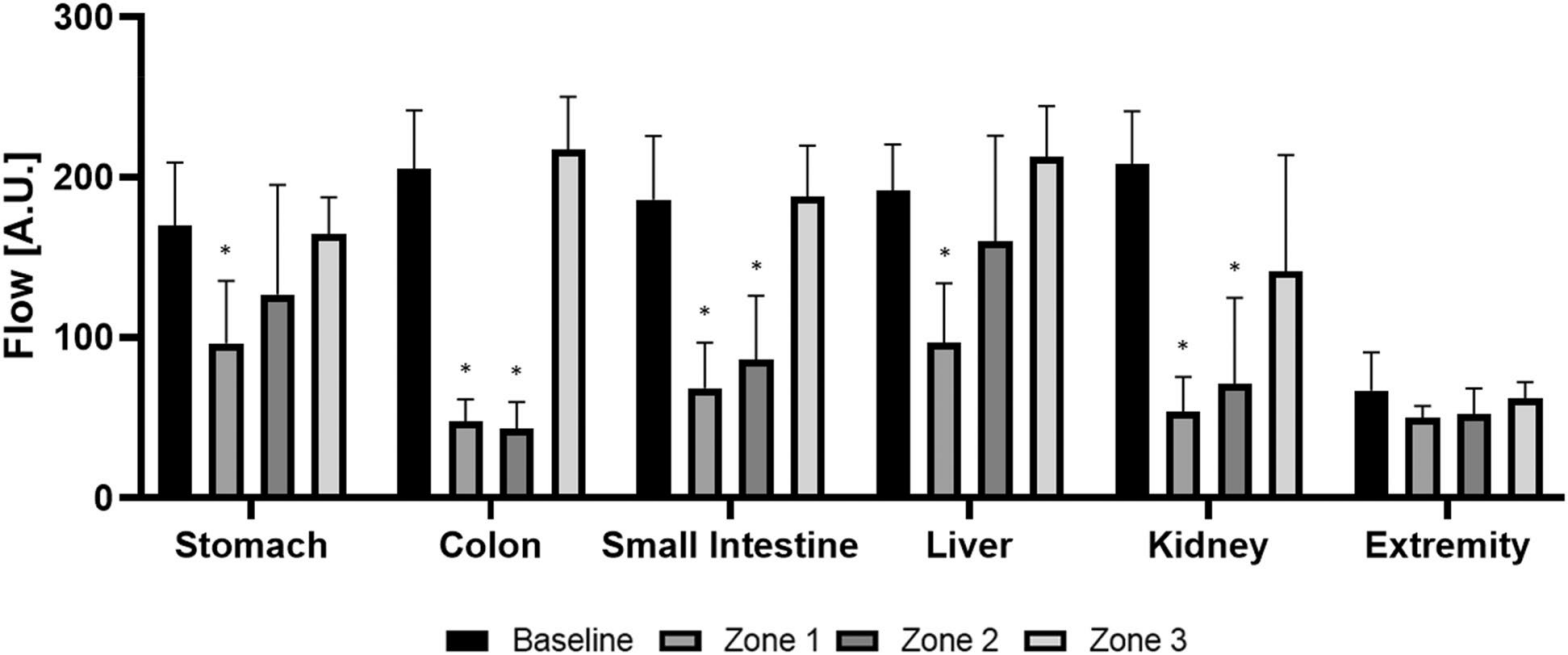
Comparing local microcirculation (flow rate in arbitrary units, A.U) at the parenchyma of each organ. Flow at the stomach and liver decreased significantly during occlusion in zone 1, no changes occurred during occlusion in zones 2 or 3. Flow at the colon and small intestines, and the kidney decreased significantly during occlusion of zones 1 and 2, when compared to baseline, but not during occlusion in zone 3. Flow at the extremity did not change significantly. * Significantly different when compared to baseline (*p* < 0.05)

The MAP proximal to the balloon was significantly increased to 171.7%, whereas the MAP distal to the balloon decreased by − 85.3% (*p* < 0.001, Fig. 1). The intra-abdominal microcirculation (flow) was significantly decreased (*p* < 0.001), (− 43.4% at the stomach, − 76.7% at the colon, − 63.2% at the small intestine, − 49.6% at the liver and − 74.3% at the kidney) at all measured sites, when compared to baseline values. Microcirculation at the extremity (− 25.4%) was not significantly altered (*p* = 0.23, Fig. 2 and Table 1). Oxygenation levels were significantly reduced in the kidney (− 98%, *p* = 0.013), and the liver (− 94.4%, *p* = 0.001).

**Table 1:** Local oxygenation was independent of the zone of occlusion at the stomach, small intestine, colon and the extremities.

Zone-dependent oxygenation (sO2 [A.U.]) of organs during occlusion of the aorta					
	Baseline	Zone 1	Zone 2	Zone 3	*p*-value
*n*	6	6	6	6	
Stomach (mean ± SD)	69.3 (21.9)	63.5 (11.4)	73.2 (10.5)	81.5 (15.5)	n.s
Small intestine (mean ± SD)	64.8 (17.3)	46.8 (8.9)	47.5 (6.8)	56.8 (5.8)	n.s
Colon (mean ± SD)	74.0 (48.8)	34.0 (19.4)	50.0 (18.7)	56.7 (22.6)	n.s
Extremity (mean ± SD)	86.7 (10.2)	65.3 (17.0)	79.5 (13.6)	74.5 (8.1)	n.s
Kidney (mean ± SD)	50.2 (8.9)	1.0 (0.0)	12.6 (25.9)	36.8 (42.0)	0.013
Liver (mean ± SD)	41.3 (15.3)	2.3 (2.2)	23.3 (35.7)	56.0 (6.9)	0.001

Local oxygenation levels changed significantly at the kidney, and the liver during occlusion in zones 1 and 2.  ± : Standard deviation; n.s.:  not significant zone-dependent changes in local oxygenation

#### Changes during occlusion in Zone 2

The MAP proximal to the balloon was significantly increased to 145.1% (*p* < 0.001), whereas the MAP distal to the balloon was decreased by 84.6% (Fig. 1). During occlusion, microcirculation (flow) at the colon (− 79.0%) and small intestine (− 53.8%), and kidney (− 65.9%) were significantly decreased (*p* < 0.001), whereas the stomach (− 25.6%), liver (− 16.3%), and the extremity (− 21.9%) did not show any significant changes (*p* = 0.34, Fig. 2 and Table 1). While microcirculation of the extremity was independent of the zone of occlusion, microcirculation in intra-abdominal organs generated zone- and organ-dependent changes (Fig. 2 and Table 1). Oxygenation was significantly reduced only in the kidney (− 74.9%, *p* = 0.01), whereas oxygenation in the other intra-abdominal organs, and the extremity were not significantly altered (*p* = 0.26).

#### Changes during occlusion in Zone 3

The MAP proximal to the balloon was significantly increased by + 10.3% (*p* < 0.001), whereas the MAP distal to the balloon was decreased by − 67.7% (Fig. 1). Occlusion did not affect microcirculation in intra-abdominal organs, or the extremity. Zone-dependent changes are summarized (Fig. 2 and Table 1). Oxygenation levels were not significantly altered in the intra-abdominal organs or the extremity (*p* = 0.48).

#### Post-interventional alterations

**Fig. 3:**
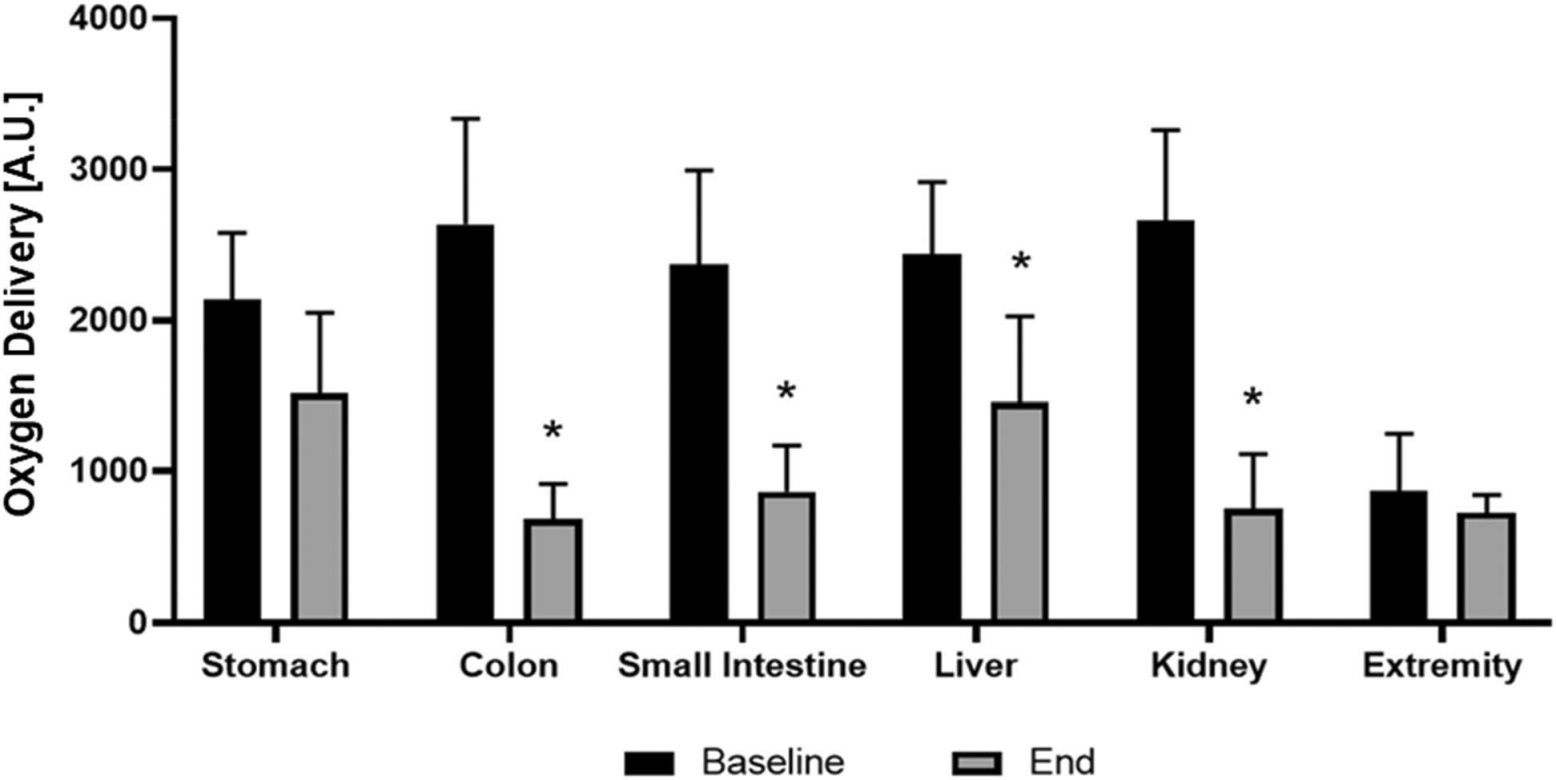
Oxygen delivery at baseline and experimental end. Oxygen delivery decreased significantly at the colon, small intestine, liver and kidney at experiment end. Oxygen delivery was comparable to baseline values at the stomach and the extremities. * Significantly different when compared to baseline (*p* < 0.05)

Lactate levels were significantly increased from 0.85 mmol/l (± 0.5 mmol/l) at baseline, to 5.14 mmol/l (± 3.2 mmol/l) (95% CI − 4.7 to − 3.8, *p* < 0.001) at experiment end. Oxygen delivery was significantly affected in the colon (− 74.2%), small intestine (− 63.7%), and the kidney (− 71.7%) (*p* < 0.001), but not at the stomach (− 29.6%), liver (40.5%), or the extremity (− 16.5%) (Fig. 3).

## Discussion

REBOA is an endovascular balloon used to limit blood loss during severe hemorrhage in severely injured patients [18]. This study described and compared local microcirculatory changes during occlusion of the aorta, at occlusion zones 1, 2, and 3. The main observations are:

1. Physiological flow rates and local microcirculation at baseline were organ-specific and differed significantly between intra-abdominal organs and the extremity.
2. Microcirculatory changes were organ specific following the same occlusion of the aorta: Circulation at the colon was more severely affected (− 76.7%) compared with the stomach (− 43.4%).
3. REBOA occlusion was associated with diverse microcirculatory responses in intra-abdominal organs;
  - Occlusion at Zone 1: All intra-abdominal organs demonstrated significantly decreased microcirculation (flow, SO_2_), the MAP proximal to the occlusion peaked most.
  - Occlusion at Zone 2: Only the colon, small intestine, and kidney showed a significantly decreased microcirculation, whereas microcirculation at the stomach, liver and the extremity showed no significant changes.
  - Occlusion at Zone 3: No organs displayed significant alterations in microcirculation (flow, SO_2_), when compared to baseline levels.

Our study showed that abdominal organs had different baseline microcirculatory values (flow and SO_2_) when compared to each other. It is accepted that the specific functional activity of organs is affected by changes in local microcirculation [19]. While our animals were under general anesthesia in a resting state, the musculature of their peripheries were likely to be less perfused, when compared to intra-abdominal organs. Organ specific differences in microcirculation are well known. Anesthesia and sedativa have an additional impact on organ-specific microcirculation [20]. This explains the organ-specific baseline values in local microcirculation in our study. Significant flow rate changes were observed in the colon, small intestine and kidney, while changes at the stomach and the extremities were less pronounced. Previous studies have shown that intra-abdominal organs react more sensitively to hemorrhagic shock after polytrauma [21]. Restoring perfusion of intra-abdominal organs may reduce complication rates in severely injured patients with hemorrhage [22].

This study is the first to demonstrate an organ specific intensity of local microcirculation changes after REBOA. It appeared that the changes of microcirculatory flow at the colon and small intestine to REBOA were more severe when compared to the stomach or liver. It has been shown that the colon is more sensitive to blood flow alterations, as it affects gut-barrier homeostasis [23, 24], a process that has not been observed and described at the stomach. This might indicate the colon to be the limiting factor of occlusion time during REBOA. The potential disruption of the gut-barrier might be a risk factor for bacterial translocation and further severe complications. The organ-specific microcirculatory changes may also contribute to the increased vulnerability of intra-abdominal organs, when compared to the extremities [25]. While microcirculation in the intra-abdominal organs reacted more severely to the occlusion, the local microcirculation at the extremities was not affected during the short-term occlusion.

Zone-dependent occlusions appear to influence perfusion of each organ, in a specific manner. Occlusion in Zone 1 decreases perfusion distally from the left subclavian artery [26]. We, however, observed that the occlusion did not cause a complete stop in intra-abdominal organ perfusion. The liver, as a main targeting organ, showed a 49% decrease in microcirculation during complete occlusion in Zone 1. This observation questions complete occlusion, since it has been shown that intermittent REBOA is potentially superior to complete REBOA, as the former is associated with improved survival in solid organ injuries [27]. Similarly, retrograde flow is also discussed as a possible mechanism for visceral perfusion [28]. This concept is key to avoiding inflation of the balloon in zone 2 [28, 29]. Our study revealed that visceral organs proximal to the balloon occlusion-zone were not affected by decreased microcirculation, blood flow, or oxygenation. The occlusion in Zone 2 affected the microcirculation with organ-specific severity. While the colon exhibited the highest microcirculatory changes, the small intestine and kidney were less severely affected. It has been described, that during hemorrhagic shock, physiological systems redistribute perfusion to vital organs [30, 31]. This redistribution (central sparing) leads to a more severe decrease in colic perfusion, when compared to renal perfusion.

REBOA in Zone 3 neither affected the intra-abdominal organs, nor microcirculation at the lower extremity. Blood flow at the lower extremity appeared to be independent of short-term REBOA regardless of the zone, at least in pigs. As previously stated, one reason for this observation may lie in the baseline redistribution of blood flow, based on organ specific functions and needs during the experiment [19]. The decreased baseline flow rate may falsely lead to comparable flow values during aorta occlusion. Occlusion of the distal aorta did not affect collateral vessels that perfuse the muscles of the lower extremity. Mini-pigs have a sufficient collateral system that is capable to cover immediately for an acute infrarenal aortic occlusion; these collaterals are not as well developed in humans [11, 32]. One study investigated the complete disruption of blood flow of the lower extremity using a tourniquet in humans, and found that after 60 min, no associations were found with higher rates of limb loss after lower extremity arterial trauma [33]. It appears that blood reserves and collateral vessels maintain a certain flow during hemorrhage or blood flow disruption. These collaterals may increase the time of balloon occlusion, without increased risk of muscle necrosis.

### Study strengths and limitations

The microcirculation (O2C) measurement device was validated and used in several other studies [34, 35]. One may argue that occlusion for ten minutes may have been too short to observe changes in microcirculation, especially in the extremity. We also showed that even a relatively short occlusion time of the aorta was associated with substantial decreases in organ perfusion. The experiment setting involved a laparotomy with direct measurements of different organ microcirculatory changes, during and after REBOA. However, in clinical practice, insertion of a balloon for severe bleeding is usually performed with a closed abdomen [36]. Thus, the influence of potential intra-abdominal hypertension during reperfusion after zone-dependent REBOA was not be evaluated. This experiment aimed to analyze short-term microcirculatory changes in different organs during REBOA. The 10-min ischemia–reperfusion time might not be consistent with clinical practice, and the animals were stabile during the experiment, which further might not simulate the clinical situation that might indicate the use of REBOA. However, the data provided by this study potentially guide future investigation: the colon reacts more severely to the same occlusion as the stomach does. Future investigations should aim to the time-point of irreversible colon-damage after REBOA, since the colon reacts most sensitive to occlusion. Second, based on the present results, it becomes evident, that the effect of REBOA depends on zone and organs. Thus, this study might guide future investigations to focus on the effect of REBOA on organs of interest. Lastly, the stable condition of the animal might not represent the clinical situation, however, this study aimed to analyze the zone- and organ specific effects of REBOA Therefore, we discarded hemorrhagic shock or unstable situation in order not to mask the effects or REBOA by additional injuries or hemorrhage. Further investigations are warranted to address microcirculatory changes during hemorrhage and REBOA. The repeated occlusion and release of perfusion might introduce a pre-conditioning effect. This effect could alter the microcirculatory measurements. However, we aimed to minimize the pre-conditioning effect by choosing a short ischemia/reperfusion time of 10 min. This I/R time is long enough to measure microcirculatory changes, but short enough to prevent potential irreversible circulatory damages and to minimize the risk of pre-conditioning effect. Yet this cannot be ruled out completely.

## Conclusion

This study revealed that REBOA is associated with both zone- and organ-specific changes in microcirculation. While perfusion of the extremities was unaffected by 10 min REBOA, microcirculation in intra-abdominal organs was diminished. The microcirculation of the colon was found to be most vulnerable to disruption of microcirculatory homeostasis upon REBOA when compared to other intra-abdominal organs.

## Figures and Tables

**Fig. 4:**
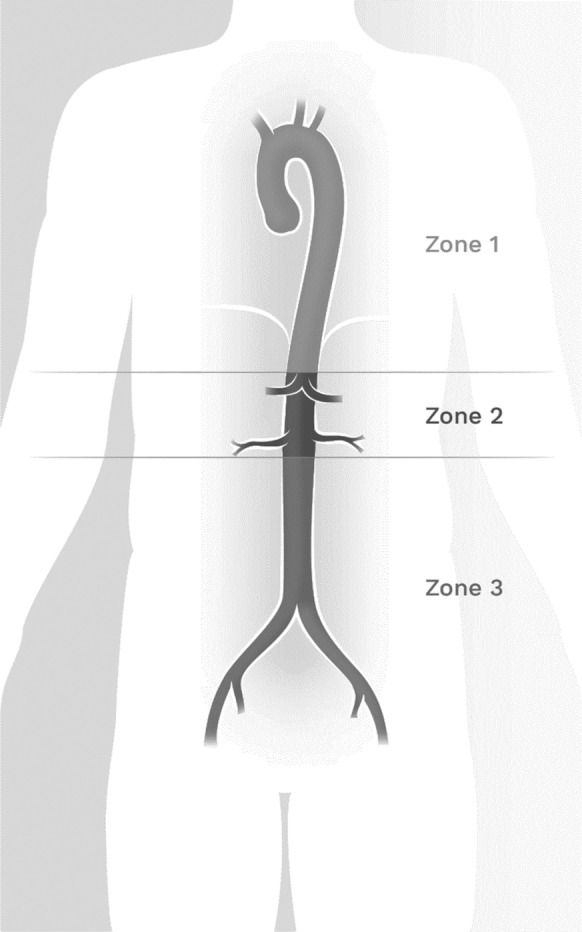
Scheme of aorta and the anatomic location of each zone: Zone 1: Descending aorta to diaphragm; Zone 2: Between celiac trunk and the lowest renal artery; Zone 3: Distal from lowest renal artery to iliac branch.

## Data Availability

All data and materials are available upon reasonable request.

## Appendix

See Fig. 4.
